# Mouse Models for Human Herpesviruses

**DOI:** 10.3390/pathogens12070953

**Published:** 2023-07-19

**Authors:** Ivana Kutle, Anne Dittrich, Dagmar Wirth

**Affiliations:** 1 Research Group Model Systems for Infection, Helmholtz Centre for Infection Research, 38124 Braunschweig, Germany; kutle.ivana@mh-hannover.de (I.K.); anne.dittrich@inscreenex.com (A.D.); 2 Institute of Experimental Hematology, Hannover Medical School, 30625 Hannover, Germany; 3 InSCREENeX GmbH, Inhoffenstraße 7, 38124 Braunschweig, Germany

**Keywords:** human herpesvirus, mouse models, interspecies models, mouse orthologue viruses, humanized models, xenografted mice, review

## Abstract

More than one hundred herpesviruses have been isolated from different species so far, with nine infecting humans. Infections with herpesviruses are characterized by life-long latency and represent a significant challenge for human health. To investigate the consequences of infections and identify novel treatment options, in vivo models are of particular relevance. The mouse has emerged as an economical small animal model to investigate herpesvirus infections. However, except for herpes simplex viruses (HSV-1, HSV-2), human herpesviruses cannot infect mice. Three natural herpesviruses have been identified in mice: mouse-derived cytomegalovirus (MCMV), mouse herpesvirus 68 (MHV-68), and mouse roseolovirus (MRV). These orthologues are broadly used to investigate herpesvirus infections within the natural host. In the last few decades, immunocompromised mouse models have been developed, allowing the functional engraftment of various human cells and tissues. These xenograft mice represent valuable model systems to investigate human-restricted viruses, making them particularly relevant for herpesvirus research. In this review, we describe the various mouse models used to study human herpesviruses, thereby highlighting their potential and limitations. Emphasis is laid on xenograft mouse models, covering the development and refinement of immune-compromised mice and their application in herpesvirus research.

## 1. General Considerations for the Development of In Vivo Models for Human Herpesviruses

Human herpesviruses are wide-spread large double-stranded DNA viruses (for recent reviews on viral entry and replication see [1,2,3,4,5]). They belong to the large family of Herpesviridae and are categorized into alpha-, beta- and gammaherpesvirinae subfamilies. The different subfamilies share specific characteristics like cellular tropism or replication dynamics after infection. Due to their high prevalence worldwide and the ability to cause serious illnesses combined with limited treatment options, they pose a significant global health risk [6]. Table 1 summarizes the nine human herpesvirus species and gives an overview of their tropism, associated pathophysiology, and representative orthologues in animals.

When herpesviruses infect healthy individuals, the infection is immediately counteracted by various host defense mechanisms. These comprise both early (innate) and late (adaptive) responses, which together can usually control virus replication and spread [7,8]. However, viral clearance has not been reported [9], and a latent, lifelong state of persistent infection is established. In this latent state of infection, viral transcription is reduced to a level that maintains the viral genome in the infected host without generating detectable levels of new viral particles [10,11,12].

While acute infections usually do not pose a particular problem in healthy individuals, severe consequences can arise when the immune system is compromised, e.g., in transplant patients. In this case, viral replication and dissemination are not sufficiently controlled, which can result in organ failure and death. Another health issue can arise from the gammaherpesviruses Epstein–Barr virus (EBV) and Kaposi’s associated herpes virus (KSHV), which are tumorigenic and can unfold an oncogenic potential in latent phases of infection [9,13,14].

Like all herpesviruses, the human herpesviruses are largely species-specific. This host-specificity represents a challenge for investigating human herpesviruses in vivo, which fostered the development of a variety of experimental in vitro model systems. Besides comparably simple in vitro cultures of primary and immortalized human cells [15,16,17], advanced co-culture systems, tissue cultures [18,19], and ‘organs-on-a-chip’ [20,21] have been exploited to unravel the molecular mechanisms of herpesvirus infections and improve drug development for alphaviruses [22,23], betaherpesviruses [24,25] and gammaherpesviruses [15,16,17]. These culture systems represent valuable models to investigate the basic principles of virus/cell interactions. However, many challenges in herpesvirus research, such as the development of successful antiviral drugs and novel therapies, require the monitoring of infection dynamics and immune responses on a systemic level. To this end, an adequate in vivo environment closely reflecting the conditions in infected human individuals is essential. Recently, various animal models have been employed to mimic human herpesvirus infections. These models range from small animals like mice and rabbits to large animals such as pigs and primates [26,27], and some of them are listed as examples in Table 1. In this review we focus on mouse-based models.

Since herpesviruses colonized mammals before the divergence of rodents and primates, the evolution of herpesviruses coincided with the evolution of the mammalian hosts. As a result, the known herpesviruses are characterized by pronounced species-specificities. While basic virologic principles are maintained within the subfamilies from different species, various phenotypic differences arose from specific viral and host factors interactions. These concern not only viral entry but also various interactions post-entry that are crucial for the viral life cycle. This includes maintenance, replication, and immune interactions. Consequently, herpes viruses usually have a restricted tropism and cannot infect and/or productively replicate in other species. Moreover, the codivergence of herpesviruses and their host is reflected by the emergence of a number of unique genes specific for a particular virus and not present in virus homologs infecting other species, so-called ‘private’ genes [28].

To study herpesvirus infection in vivo, mice are of particular interest. Due to their close relation to humans, small size, and high reproduction rate, mice represent an economical and well-established animal model. Robust procedures for genetic modifications provide access to an ever-growing number of knock-out and mutant mouse strains, allowing the investigation of mouse genes in the context of infection.

In the last few decades, different types of mouse models have been established to investigate human herpesviruses. Interspecies models have been established for HSV-1 and HSV-2, given that these human viruses can infect the mouse. Moreover, the mouse orthologue herpesviruses MCMV, MHV-68, and mouse roseolovirus (MRV) allow the investigation of infection in the natural host, thereby providing coherent virus/host systems (Table 2). While these two types of models broadened the understanding of herpesvirus infections, they are hampered by the fact that the interaction between virus and host is not native (in the case of the interspecies models) or does not fully reflect infections in humans (in the case of the mouse orthologues). To close this gap, xenotransplantation models have been developed in recent years in which human cells or tissues are functionally integrated into immunocompromised mice and subsequently used for infection with human herpesviruses. Table 3 summarizes the experimental models for the most relevant human herpesviruses. In the following two chapters, we briefly summarize the interspecies mouse models based on HSV and the mouse orthologue virus models. Subsequently, we will discuss the development of xenograft models and highlight the application of these models to investigate herpesvirus infections.

## 2. Mice Infected with Promiscuous Human Alphaherpesviruses

Among the various human herpesviruses, the human herpes simplex viruses 1 (HSV-1) and 2 (HSV-2) are unique in their ability to productively infect mice. This makes the mouse a valuable interspecies infection model for HSV. In humans, many infections with HSV remain unnoticed. Relevant consequences, however, result from HSV infection in the eye ranging from conjunctivitis and retinitis to severe herpes stromal keratitis that might end in blindness. Furthermore, HSV can be transmitted perinatally from mother to child and causes neonatal infection with high morbidity and mortality rates when untreated [36]. Neuronal cell infection can induce severe diseases like herpes simplex encephalitis (HSE) in the central nervous system [37]. Additionally, in HSE patients, a link between HSV infection and Alzheimer’s disease is being investigated [38]. Delayed diagnosis and treatment can result in neurological damage or even death [39]. In humans, HSV establishes infection in epithelial cells, keratinocytes, and fibroblasts at mucous sites. HSV glycoproteins B and C generally attach to heparan sulfate components. Cell entry requires binding all viral glycoproteins to the cellular surface receptors HVEM (herpes virus entry mediator), nectin 1, or nectin 2, which induces plasma membrane fusion or endocytosis [40,41,42]. Upon infection, HSV further propagates in the sensory cells of the peripheral and central nervous systems. The variety of cellular tropism within the host offers a broad range of potential sites for infection. A major benefit of mouse models is that several cellular entry receptors needed for the infection of HSV-1 and HSV-2 are shared.

In mice, HVEM and nectin-1 are both expressed on epithelial cells and fibroblasts [43,44]. HSV-1 can infect various mouse tissues, including the eye, skin, and mucosal membranes. By applying a low dose of virus to a small scratch on the surface of the eye a localized infection is established, which reflects natural infection in humans. In contrast, intraperitoneal injection results in systemic infection. HSV-1 infection in mice targets the same types of cells as in humans, primarily epithelial cells and nerve cells. Exploiting the various mouse knock-out strains, HSV-induced immune responses, neuroinvasion as well as latency have all been investigated [39,45]. This demonstrated the crucial role of nectin-1 for the infection of neurons and the establishment of HSE [46]. 

After initial replication, HSV-1 establishes a latent infection state in nerve cells, where the virus remains dormant until it is reactivated. In both humans and mice, the virus can infect sensory neurons, including the trigeminal ganglia in the face and the dorsal root ganglia in the spinal cord. The establishment and maintenance of HSV latency in mice share several similarities with human infection, making mice a valuable model for studying the virus’s tropism and pathogenesis in a relevant host system [47]. However, when HSV latency and reactivation are investigated in mice, a limiting factor is the lack of frequent spontaneous reactivation of HSV infection [48].

HSV-2 infection can also be established in mice, although mice are less susceptible to HSV-2. HSV-2 infection in mice can cause similar symptoms to those observed in humans. However, the severity and presentation of symptoms may vary depending on the mouse strain, the dose and route of viral inoculation, and other experimental conditions. After the acute phase of infection, HSV-2 can establish latency in sensory neurons without any clinical signs of infection. However, the virus can reactivate under certain conditions from latency, leading to recurrent infections and associated clinical signs [49,50].

It is important to note that, like in other herpesviruses, there may also be differences in the immune response and pathogenesis of HSV infection between mice and humans. Therefore, caution should be exercised when extrapolating results from mouse models to human HSV infection. Especially in the context of CNS infection, differences are observed in terms of clinical pictures and symptoms. Sehl and colleagues established an improved mouse model for HSE based on a pig alphaherpesvirus that could mimic the histopathological changes and inflammatory responses as it was observed in humans [51]. It remains to be shown if this model can contribute to understanding this disease.

Apart from herpes simplex viruses, other family members cannot infect and/or propagate in mouse cells. The underlying mechanisms are only partially understood and appear to be multifaceted. Finnen and co-workers demonstrated that VZV can enter Chinese hamster ovary cells, initiate the viral life cycle, undergo replication, and produce at least one late structural protein (gE). However, there is no evidence for viral progeny production, suggesting that a late step is impaired [52]. In a recent study, Reynolds and colleagues succeeded in making mice susceptible to HHV-6. To this end, they expressed the HHV-6 entry receptor, human CD46, on murine brain glial cells. After intracranial injection of HHV-6A, viral DNA was detectable in the brain for up to 9 months post-infection, and the infected glial cells produced pro-inflammatory cytokines ex vivo [53]. However, genetic strategies that render mice competent for human herpesviruses require the knowledge of relevant steps of the viral life cycle impaired in mouse cells and the identification of the contributing factors. So far, however, knowledge in this field is still limited. While previous studies identified the inability of MCMV to block apoptosis as a main reason for the failure of productive infection in human cells [54], HCMV infection of mouse cells is blocked at the immediate early stage [55,56]. A recent study indicated that the knock-down of cellular ND10 enhanced HCMV protein production in mouse cells, while the block in viral replication was not overcome [57]. Thus, more research is required to elucidate the roadblocks of replicative human herpesvirus infection in mice on a molecular level.

## 3. Mouse Herpesvirus Orthologues as Models for Beta- and Gammaherpesviruses

Murine cytomegalovirus (MCMV), murine herpesvirus 68 (MHV-68), and murine roseolovirus (MRV) are natural mouse pathogens of the beta- and gammaherpesvirus subfamilies, respectively. These viruses share the basic virologic mechanisms and features with their human orthologues. They are of particular value in investigating viral dissemination, viral protein interactions with host factors, viral pathogenesis, and the immune response in a living animal. The following subchapters summarize the relevant features and recently highlighted benefits of the mouse herpesvirus orthologues in vivo.

### 3.1. MCMV as a Model for HCMV

MCMV shares many features with HCMV [58]. Similar to the human counterpart, MCMV infects a broad range of cells in vivo, and infections usually occur without relevant manifestations. In experimental mouse models, various routes of infection are used, such as intranasal, intraperitoneal, intravenous, or via the footpad. Notably, dissemination of MCMV involves defined myeloid subsets that are specific for the particular route of infection [59].

Studies in recent years provided valuable knowledge concerning viral mechanisms [60] and deep insights into immune responses [61]. These studies have elucidated a plethora of virus–host interactions underlying host immune responses and virus-mediated immune evasion. Although mice and men share the general mechanisms of immune responses to cytomegalovirus, there are particular differences arising from the presence of unique HCMV-specific genes as well as the specific nature of the shared genes and the corresponding host factors, as reviewed elsewhere [60,62].

A strong focus in MCMV research was laid on identifying cell types that contribute to latency. Various studies implied that latent MCMV is localized in stromal cells and tissues rather than in the hematopoietic system [63]. Formal proof was given that MCMV latency can be established in liver sinusoidal endothelial cells [64,65]. While this does not exclude other reservoirs, interestingly, attempts to transfer infection via hematopoietic cell transplantation failed (reviewed in [66,67]). This seems to be in contrast to the human system, where latent CMV infection has been confirmed in hematopoietic cells, namely early myeloid cells and hematopoietic stem cells [62,66]. However, the limited access to tissue cells from otherwise healthy donors makes it difficult to exclude other sites of latency in humans. Thus, in the human system, it remains unknown if hematopoietic stem cells and pre-myeloid cells are the only carriers of latent viruses [67].

On the other hand, the apparent discrepancy in mouse and humans might not necessarily hint towards differences in the human versus mouse CMV infections. Rather, as Reddehase and colleagues suggest, it might also indicate different states of latency—transient latency in the hematopoietic compartment and a life-long latency in tissue cells [67,68]. In this regard, the different cell carriers of latent infections in mice and humans might reflect different foci of research in the two systems. It may also be a consequence of the rare latent infections and limited access to potentially infected cells [67].

A particular risk arises from HCMV infections during pregnancy, representing the leading cause of viral congenital disabilities with an overall prevalence of 0.67% [69]. HCMV can cross the placental barrier by infecting cytotrophoblasts, and it can be transmitted during delivery or via breastfeeding. Vertical transmission of HCMV can cause severe neuropathogenesis, with long-term sequelae such as neurodevelopmental disabilities, cerebral palsy, and hearing loss [69]. In contrast to HCMV, MCMV fails to be transmitted vertically, and thus intrauterine infections cannot be mimicked. An exception can be found with severe combined immunodeficient (SCID) mice that lack B and T cells [70]. In this model, efficient virus transmission from infected dams was reported; however, the immunosuppressed background limits the relevance of this model. To investigate the consequences of early CMV infections in immunocompetent mice, several studies used the intracranial infection of embryos during gestation or intraperitoneal infection in newborn pups (reviewed in [71]). Intracranial infections require complex experimental techniques and can be associated with high death rates. Moreover, while this technique can mimic infection of brain cells during the early stages of brain development, it cannot fully mimic the situation during human congenital infection since it lacks hematogeneous viral spread and does not include potential influences arising from previous infections of peripheral tissues. In a recent report, MCMV was transferred via intracranial infection of fetal mice at day 13.5 of embryonal development, with a consistently high frequency of pups with neurological disorders, including auditory and behavioral abnormalities [72].

More recently, another mouse model was established based on direct injection of the virus into the mouse placenta on day 12.5 of gestation when neurogenesis is active. Approximately 40% of fetuses were infected, and pups showed infection of various organs, most efficiently in the brain. Hearing deficiencies and infection of the cochlea were confirmed in offspring [15].

Intraperitoneal infection of pups emerged as an alternative experimental condition to investigate the consequences of early infection. This infection route is followed by hematogenous viral spread and viremia in peripheral organs prior to infections in the central nervous system. Notably, neonatally infected mice develop brain alterations, including focal encephalitis and neurological sequelae [73], including hearing loss [39,74,75].

### 3.2. Murine Roseolovirus (MRV) as a Model for HHV-6 and HHV-7

Murine roseolovirus (MRV, also known as mouse thymic virus, MTV) was identified as another natural mouse betaherpesvirus which was confirmed by its homology to HHV-6 and HHV-7 [76]. So far, MRV has not been broadly exploited as a model for betaherpesviruses, and thus knowledge is limited. In experimental conditions, MRV infection is mainly studied in neonates, where infection can be accompanied by significant consequences such as thymic necrosis and autoimmune diseases. After neonatal infection, the virus is detectable mainly in various immune cells of lymphoid organs and has a particular impact on the T cell compartment [77]. Early evidence was given that latency is established in mice [78], while most experimental studies on MRV focus on the lytic phase of infection. A recent study demonstrated the role of MRV in dampening central tolerance by impairing thymic selection. In the absence of ongoing infection, there was an observed development of autoreactive T cells and a broad range of autoantibodies [79].

To investigate a potential link between roseolovirus infection and Alzheimer’s disease, Bigley and colleagues used MRV to infect a transgenic mouse model in which overexpression of mutants of the human amyloid precursor protein (APP) and presenilin-1 leads to plaque formation. However, the virus-induced neuroinflammation, their study did not provide any hint that MRV infection supports deposition of the characteristic protein aggregates, thus arguing against a direct relation between viral infection and the development of Alzheimer’s disease [80].

### 3.3. MHV-68 as a Model for EBV and KSHV

MHV-68 is a gammaherpesvirus and, as such, represents a model for Epstein–Barr-Virus (EBV) and Kaposi’s associated herpesvirus (KSHV). MHV-68 shares key biological functions and encodes many homologous genes relevant for replication, such as the latency-associated nuclear antigen (LANA), viral cyclin, and viral GPCR genes. Similar to EBV and KSHV, mouse MHV-68 also infects B cells and establishes life-long latency, which is associated with lymphoproliferative disorders and the development of malignancies. However, MHV-68 fails to mimic the formation of endothelial tumors characteristic of KSHV infections. An overview of MHV-68-associated pathogenesis in mice and the interaction with the host was provided previously [81]. More recently, the features of innate and T cell responses in the context of MHV-68 infection in mice and their relevance for EBV were summarized [82].

Based on the robust establishment of persistent infections in mice, MHV-68 represents a powerful model to clarify the mechanisms underlying the establishment of chronic viral infections in vivo [83]. In this regard, MHV-68 was used to elucidate the relevance of lytic replication for the establishment of viral latency. Gupta and colleagues used cre recombinase-expressing mice to eliminate the viral ORF50 gene and thereby conditionally ablate lytic MHV-68 infection in B cells. Excision of ORF50 did not reduce MHV-68 maintenance compared to wild-type virus, demonstrating that viral replication is not necessary for the establishment of latency [39]. In contrast, based on a similar genetic system, the MHV-68 encoded latency-associated nuclear antigen LANA was found to be crucial for latency since mLANA-deficient viruses resulted in the complete loss of viral genomes [84].

Another question recently addressed is the contribution of the innate response of the host, in particular the type I interferon (IFN) response, for control of viral latency. The type I IFN response represents a first-line defense system against many viral infections and is also essential to control acute MHV-68 infection [85,86,87]. To investigate the in vivo role of the type I antiviral interferon (IFN) response to latent infection, Schwerk and colleagues employed a highly sensitive bioluminescence reporter mouse to detect low levels of IFN released upon viral reactivation. They observed increased viral dissemination when type I IFN signaling was impaired, suggesting that this defense mechanism contributes to controlling viral latency. However, despite overall latency control by IFN, rare cell-to-cell transmission of MHV-68 was observed. These arose from permanent low-level reactivation events, indicating that IFN cannot entirely prevent viral dissemination during latency [88].

### 3.4. Limitations of Orthologue Viruses as Models for Human Herpes Viruses

As a consequence of an early divergence in evolution, not all the human herpesvirus genes have matching homologs in the mouse viruses. These virus-specific genes represent the above-mentioned “private” genes. Some of these genes have a notable role in infecting and manipulating human cells. Among them, HCMV UL133-138 genes and the gene UL7 have been identified as crucial players in regulating viral latency and reactivation [89,90,91]. Mouse viruses also have unique genes, such as the secreted proteins M1, M3, and M4 of MHV-68, which are involved in latency regulation and are absent in KSHV and EBV [92,93]. Thus, mouse homolog viruses do not reflect the full genetic repertoire of human viruses and vice versa, resulting in differences in host/virus interactions and species-specific modulation of infection and immune response. The presence or absence of viral proteins can also result in differences in host interactions and modes of action. Also, variations in protein sequences can account for differences.

An example is the major immediate early protein 1 (IE1) of MCMV, which does not bind to chromatin like HCMV IE1. The functional consequences of this interaction are still not completely clear [94]. Similarly, the above-discussed differences in cellular reservoirs of latent CMV infections in mice and men might be a consequence of species specificities.

Together with the fact that most human herpesviruses do not infect mice, all these differences represent a constant limitation in modeling human herpesvirus infections, resulting in a still incomplete understanding of virus dissemination in vivo, pathogenesis, host immune responses, and development of potent antivirals and vaccines. The need to investigate infection of the human viral target cells and corresponding immune responses in vivo fostered the development of various humanized mouse models with engrafted human cells or tissues.

## 4. Investigating Human Herpesviruses in Human Cell-Engrafted Mice

### 4.1. Engraftment of Functional Human Cells in Mice

#### 4.1.1. Cell or Tissue Sources for Xenografts

The wide-ranging ability of herpesviruses to infect various tissues and cells [1,7,8] presents both options and challenges for the establishment of xenograft models [1,2,4]. Considering their biological relevance for herpesvirus infections and availability, most commonly exploited human cells in murine xenograft models are hematopoietic stem cells derived from fetal tissues or cord blood.

Outside this, pieces of human tissues known to be targeted by herpesviruses, such as skin [95,96], retina [94], liver [97], or lungs [35], are regularly used for the generation of humanized mice. They offer a higher complexity since they provide different interacting cell types and maintain the tissue architecture, which can be relevant in the context of viral infections.

Yet, applying pieces of tissue or primary cells from humans not only has certain limitations in survival and persistence in mice but also generates ethical concerns. To overcome the limited availability and variability of primary human cells, infection-relevant immortalized cell lines with advanced properties were developed and used for transplantation [16,17]. However, immortalization processes and long-term cultivations frequently provoke changes in cellular behavior and biology, which limits the application of such cell lines. Innovative immortalization techniques have recently allowed the preservation of relevant characteristics and functions of primary cells, giving rise to cell lines with advanced properties [16,98]. These technologies may allow the use of such cells as a standardized, unlimited source for transplantation in the future.

#### 4.1.2. Historical Overview of the Development of Humanized Mice

The major barrier to the engraftment of human material in mice is robust xenograft rejection that is mediated by the murine complement system, neutralizing antibodies, innate immune cells (e.g., natural killer (NK) cells, macrophages, and neutrophils), and specific T cell-mediated immune responses. Thus, the first challenge towards humanized mice was to generate immunodeficient animals that are amenable to the long-term engraftment of human cells.

The development of humanized mice started more than 50 years ago and relied on spontaneously generated mouse strains deficient in various immune functions. It has progressed through several phases, each building upon the previous breakthroughs (see [99,100] for recent reviews). The earliest studies on xenotransplantation relied on nude mice, which have a defect in the Foxn1 gene encoding a DNA binding transcription factor. These animals are characterized by hairlessness and impaired normal thymus development, leading to the deficiency of mature T lymphocytes [101]. However, this model still has other functional immune cells, including B and NK cells, which can limit the efficient engraftment of human cells [102]. A breakthrough in the field was made several years afterward with the discovery of SCID mice. SCID mice contain a spontaneous mutation in the gene encoding the catalytic subunit of the DNA-dependent protein kinase (*pkrdc*), resulting in deficient T and B cells [103]. The first humanized mouse models, based on SCID mice, involved the engraftment of human peripheral blood leukocytes (SCID-hu-PBL model) intravenously or intraperitoneally [29] and transplantation of human fetal thymic and liver tissues (SCID-hu-Thy/Liv model) under the kidney capsule [30], thereby providing progenitor/stem hematopoietic cells. The main limitations of these models comprise a lack of long-term human cell engraftment (SCID-hu-PBL model), poor distribution of human cells among organs in the mouse (SCID-hu-Thy/Liv model), low diversity of engrafted cell types, and an overall failure in the generation of human-based immune responses [104,105].

Moreover, high levels of host NK cells and spontaneous generation of functional mouse T and B cells were frequently observed, which limited the engraftment of the human hematopoietic compartment [106]. Additionally, the fact that the *pkrdc* mutation also involves general defects in DNA repair and an increase in radiosensitivity, the value of this model is limited [107]. Several attempts were made to improve the engraftment of human cells in SCID mice. Among them, the crossing of SCID mice to beige mice, which exhibit NK cells with decreased cytotoxic activity, represented an important milestone. Grafting efficiency is significantly improved in SCID/beige mice [108].

With the development of genetic engineering methods, targeted mutations became the ultimate way to rationally develop humanized mouse models. One of the first strategies was targeted inactivation of the recombination-activating genes (Rag) 1 and Rag2. Mice with inactive Rag1 or Rag2 genes cannot develop functional T and B cells but do not show leakiness or radiosensitivity. Still, the inherent NK cell activity in these mice limits the engraftment of human hematopoietic stem cells (HSCs) [109,110].

A significant improvement came with the introduction of *pkrdc* mutation to the background of non-obese diabetic (NOD) mice, which are characterized by reduced NK cell activity, immature and less functional macrophages, and antigen-presenting cells as well as the absence of circulating complement factors [111]. Breeding of NOD and SCID mice triggered the neoteric NOD-SCID mouse strain generation. These mice could remarkably improve the compatibility of the human immune system, which was driven by the reduction of innate immunity through the defective levels of NK and myeloid cell function [106]. NOD-SCID mice support higher levels of engraftment with human peripheral blood mononuclear cells (PBMCs) [112] and HSCs [113], as well as lower NK cell activity than any of the other strains developed before [112]. Although the development of NOD-*SCID* mice brought improvements in human cells engraftment, the use of humanized NOD-*SCID* mice as a model for human immunity remains limited by their relatively short life span, the residual activity of NK cells and other components of innate immunity, which hampers the engraftment of the human lymphoid compartment.

Many recent achievements in the field were facilitated by the knock-out of the interleukin-2 receptor (IL-2R) γ-chain gene (*Il2rg*; also known as the common cytokine-receptor γ-chain, γc) [114]. The γ-chain is a crucial component of the high-affinity receptors for a group of cytokines, including IL-2, -15, and -21, among others, which are required for the differentiation of various hematopoietic cell types [115]. The absence of the IL-2R γ-chain leads to severe impairments in T and B cell development and completely prevents NK cell development [116,117]. IL2rg knock-out mice ensure better engraftment of human tissue, HSCs, and PBMCs compared with all previously developed immunodeficient mouse models. However, a breakthrough was achieved by combining the IL2rg knock-out with either the *pkrdc* mutation or the Rag knock-out: this generated three severely immunodeficient mice strains, NSG (NOD.Cg-PrkdcscidIL2rgtm1Wjl), NOG (NOD.Cg-PrkdcscidIL2rgtm1Sug), and BRG (Balb/c Rag2−/−IL2rg−/−) [106]. More recently emerging strains such as the family of the highest immunodeficient models, as NPG (NOD-PrkdcscidIL2rgnull) and NCG (NOD-Prkdcem26IL2rgem26Nju), have been widely recognized for exhibiting a significant improvement in the rate of human engraftment. They have since become the most frequently used models in the modeling human immune system functions in health and disease [104].

The murine strains commonly used for the development of humanized murine models are described in more detail in Table 4.

**Figure 1:**
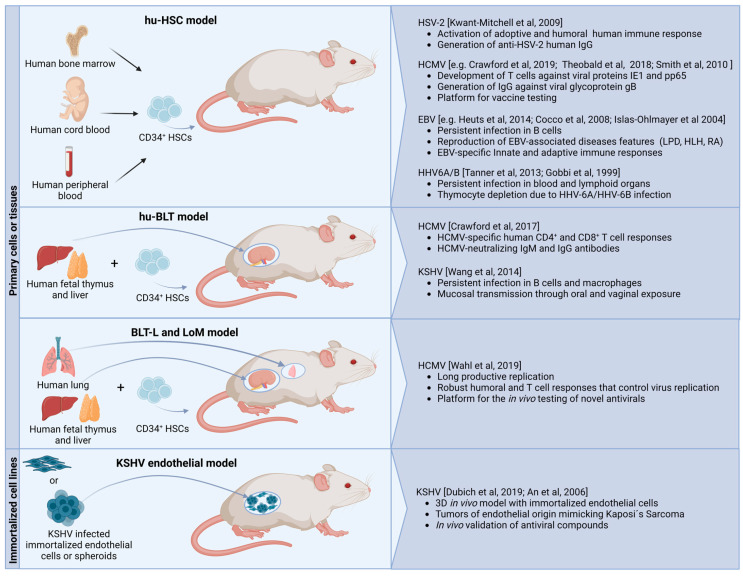
Summary of relevant mouse models for studying herpesviruses. References for the representative hu-HSC mouse models: HSV (Kwant-Mitchell et al., 2009 [122]; HCMV (Crawford et al., 2019 [123]; Theobald et al., 2018 [124]; Smith et al., 2010 [125]); EBV (Heuts et al., 2014 [126], Cocco et al., 2008 [127], Islas-Ohlmayer et al., 2004 [128]; HHV6A/B (Tanner et al., 2013 [129], Gobbi et al, 1999 [130]; huBLT model: HCMV (Crawford et al., 2017 [131]); KSHV (Wang et al., 2014 [132]); BLT-L and LoM model: HCMV (Wahl et al., 2019 [35]; KSHV endothelial model: Dubich et al., 2019 [15], An et al., 2006 [17]. The figure was created with BioRender.com (accessed on 14 July 2023).

In the following sections, we will give an overview of three main strategies in developing humanized murine models to study human herpesviruses, which we classified by the source of transplanted human material and the absence or presence of human immune system reconstitution. Figure 1 provides an overview of the relevant models. Furthermore, we will discuss the main achievements these models offered in understanding viral pathogenesis, latency mechanisms, reactivation, and development of vaccines and antivirals.

### 4.2. Herpesvirus Infection of the Hematopoietic Compartment in Xenografted Mice

The transplantation of the HSCs from the umbilical cord blood, peripheral blood, or fetal liver into immunodeficient mice gives rise to a broad diversity of blood cells that can repopulate different organs [114]. Transplantation of HSCs in immunosuppressed mice enables long-living engraftment of hematopoietic cells and activation of human adaptive immune responses (reviewed in [133]). Since various hematopoietic cells are important targets for herpesvirus infection and play a role in the establishment of latency in humans, immunodeficient mice grafted with HSCs represent valuable models for human herpesviruses. Recent studies, however, employ HSC xenografts with other cell types to better reflect the complex situation in humans. These studies will be discussed in Section 4.4. 

#### 4.2.1. HSV-1 and HSV-2 in Mice Engrafted Human Hematopoietic Cells

Due to the ability of human alphaherpesviruses to establish a productive infection in small rodents, humanized mouse models are not often used to study this viral subfamily. However, there are few reports on modeling infections of alphaherpesvirus representatives in humanized mice. A recent study reported a model in which immunodeficient BRG mice were engrafted with human umbilical cord-derived HSCs and subsequently infected with attenuated HSV-2 by intravaginal inoculation. The primary infection of murine vaginal cells with HSV-2 resulted in the activation of human T cells, which could be isolated from lymphoid organs and the vaginal tract, showing the successful establishment of an adaptive cellular response. The humoral response was also activated, and human IgG antibodies specific to HSV-2 were detected. Notably, immunized mice challenged with a lethal dose of HSV-2 showed significantly better survival in comparison to the mice without transplantation of human immune cells [122]. This model enabled studying protective human immune responses in vivo using a small animal and may become an essential preclinical tool for vaccine development for HSV-2.

#### 4.2.2. HCMV in Mice Engrafted Human Hematopoietic Cells

Contrary to alphaherpesviruses, the restricted species tropism of HCMV has driven the development of numerous humanized murine infection models (reviewed in [134]). Early humanized mouse models were based on human thymus and liver fetal tissue grafts that were successfully infected with HCMV. However, these models were limited by the lack of viral dissemination. Moreover, poor engraftment and low numbers of human myeloid precursor cells as a main reservoir of latent HCMV often failed to establish latency and monitor reactivation [135,136]. That was a driving force to develop more advanced models that contain both a reconstituted human immune system and other infection-relevant cells and tissues.

To improve engraftment and enable studying HCMV’s influence on immune response upon infection, Crawford and colleagues created a humanized mouse model reconstituted with CD34^+^ hematopoietic progenitor cells (HPCs) and matched human fetal liver and thymus tissue, the so-called huBLT model [131]. The model provided systemic reconstitution of diverse functional human hematopoietic cells and human thymic epithelium. These mice were then intraperitoneally injected with HCMV-infected fibroblasts that served as a viral inoculum. As a result, HCMV-specific human CD4^+^ and CD8^+^ T cell responses and HCMV-neutralizing IgM and IgG antibodies were generated. Despite achievements in modeling the HCMV-specific immune response in vivo, this model does not provide an appropriate time window to monitor T and B cell maturation since the complete maturation process takes more time than the experimental duration of this study [137]. Furthermore, human fetal tissue transplantation raises ethical concerns.

Several models do not require embryonic tissues. To provide a source of human myeloid precursors, a humanized mouse model has been generated by engrafting human CD34^+^ HSCs from cord blood into the bone marrow of NSG mice [125]. Eight weeks after successful engraftment, when the population of human monocytes in mice represented around 50% of the monocyte population found in healthy human PBMCs, the animals were injected with HCMV-infected fibroblasts via the intraperitoneal route. Notably, viral DNA could be detected in all organ tissues populated with human hematopoietic cells, whereas no expression of lytic viral proteins was detectable, suggesting a latent infection state.

In preparation for bone marrow transplantation in humans, donors usually receive G-CSF to mobilize stem cells into the blood. Nevertheless, the transplantation of G-CSF-mobilized stem cells originating from HCMV-positive donors is also associated with a higher risk of late-onset HCMV disease and chronic graft-versus-host disease (GvHD) in the recipients. To closer examine the clinical situation and understand the impact of G-CSF on HCMV transmission and reactivation, Smith and coworkers engrafted mice with HSCs, injected infected human fibroblasts, and treated them with G-CSF. Expression of early and late viral genes could be detected in multiple organs suggesting that G-CSF treatment provoked reactivation and viral spread [125]. This mouse model allowed the examination of HCMV infection in myeloid cells and provided a platform to investigate the factors that trigger the reactivation of HCMV from latency. Several subsequent studies further supported these findings by utilizing G-CSF treatment to simulate viral reactivation in mice undergoing HSC transplantation [91,123,138,139,140,141].

Theobald and colleagues established an alternative model with reconstitution of the human immune system in the NRG mouse strain, based on the transplantation of human CD34^+^ cells purified from cord blood. Human fibroblasts were infected in vitro with an engineered HCMV strain encoding Gaussia luciferase. These cells were subsequently injected intraperitoneally into animals and served as infected tissue. This approach ensured the long-term development of functional and mature T and B cell responses for more than 30 weeks. Moreover, the application of the reporter virus enabled following the viral infections in vivo. Finally, treatment of infected animals with G-CSF induced the reactivation of infection, which was confirmed by PCR in human CD14^+^, CD169^+^, and CD34^+^ cells in the different tissues and by in vivo imaging. Further analysis of T and B cells after reactivation reflected specific immune phenotypes characteristic for HCMV-infected humans, such as the upregulation of the programmed cell death (PD)-1 activation marker [124].

While a plethora of information has been gained in the last decades, the mouse models with HSC xenografts are often limited by the inefficient development and functionality of myeloid cells. This is considered a critical downside for studying HCMV infection, where precursor myeloid cells play a role in latency and reactivation.

#### 4.2.3. HHV-6A and HHV-6B in Mice Engrafted Human Hematopoietic Cells

Humanized mouse models were also developed for the investigation of HHV-6A and HHV-6B. Both representatives of the betaherpesvirus subfamily have a high tropism for CD4^+^ T cells, while HHV-6A also establishes lytic infection in CD8^+^ T cells, γδ T cells, and NK cells [142]. Although not directly related to the cause of any disease, HHV-6A is implicated in autoimmune diseases such as multiple sclerosis [143] and immunosuppression [130,144]. There are a few humanized mouse models reported for HHV-6A. One is based on SCID-huThy/Liv mice infected with HHV-6A intrathymically after transplantation of fetal liver and thymus. The viral infection led to the severe depletion of thymocytes, mainly reflected by the intrathymic progenitor T cells and the progressive destruction of the thymus tissue [130]. In line with this report, intraperitoneal injection of HHV-6A infected blood mononuclear cells or cell-free HHV-6A into immunodeficient Rag2−/−IL2rg−/− mice gave rise to detectable levels of HHV-6A DNA in blood, bone marrow, lymph node, and thymus. Significant changes in thymocyte populations with significant loss of intrathymic T progenitor cells were also observed. Both models induced pronounced immunosuppression as a hallmark of infection [129].

#### 4.2.4. EBV and KSHV in Mice-Engrafted Human Hematopoietic Cells

To study oncogenic EBV and—to a lesser extent—KSHV infections in HSC-engrafted mice, significant efforts have been applied to model the antiviral host immune response and infection-associated tumorigenesis. While early work in the EBV field was performed in the SCID-PBL model, the focus eventually shifted to models with a reconstituted human immune system based on NSG, NRG, or BRG mouse strains or to the more complex BLT model [127].

Xenograft mice have shown successful infection of B cells with EBV via intrasplenic [128,145], intraperitoneal [126,146], and intravenous [147,148] injection routes and enabled the establishment of latency. In addition, the models nicely recapitulate B cell lymphoproliferative disease and EBV-driven lymphoma formation, as well as clinical features of hemophagocytic lymph histiocytosis and erosive arthritis associated with EBV infection, allowing testing of different therapies for these pathologies. While the B cell compartment is highly relevant for EBV, it would be essential to consider the involvement of human oropharyngeal epithelial cells as a relevant target in lytic replication and viral dissemination in humans. However, implanting these cells in experimental models still poses a challenge. Another pitfall arises from the fact that the efficiency and characteristics of lytic infection and reactivation of EBV in xenograft mice can vary depending on the viral strain used. For example, when mice were infected with the prototypic B95-8 EBV strain, reactivation occurred two weeks after the primary infection, and cytotoxic lymphocytes efficiently controlled lytic replication [149].

In contrast, infection with other strains, such as M81, resulted in stronger reactivation of lytic replication [150]. These findings highlight the need to carefully consider viral strain selection when studying EBV infection dynamics and reactivation mechanisms in mouse models. Further models are warranted to improve our understanding of EBV infection dynamics and the interplay between viral strains and host immune responses, ultimately advancing our knowledge of EBV-associated diseases and potential therapeutic interventions.

Only a few HSCs-engrafted mouse models have been reported to study KSHV infection. The first strategy relied on the transplantation of in vitro-infected HPCs into NOD/SCID mice. This strategy enabled persistent infection and suggested CD34^+^ HPCs as a reservoir for KSHV infection and a continuous source of virally infected cells [137]. However, it remains to be clarified if this cell type contributes to latency in humans. Later, a study by Wang in 2014 demonstrated the establishment of KSHV infection in mice previously engrafted with human HSCs. In the study, BLT mice were infected with KSHV via intraperitoneal, vaginal, or oral routes. In all conditions, viral DNA was detected in B cells and macrophages, mainly in the spleen and to a lower extent in blood and bone marrow up to 29 weeks post-infection. The establishment of infection in blood cells upon vaginal infection proved the transmission of KSHV through mucosal exposure, which reflects the physiological infection route in humans. Although lytic and latent infection markers were detected in the spleen, there was no evidence of lymphoma or Kaposi’s sarcoma (KS) [132]. This observation is in agreement with the current debate that co-infection with EBV or HIV is necessary for the appearance of KSHV malignancies (reviewed in [151]) and might also indicate the need for endothelial grafts to establish KS.

### 4.3. Herpesvirus Infection in Humanized Mouse Models Prepared from Tissue Xenografts

Mouse models engrafted with hematopoietic cells exclude herpesvirus infections of other targets such as epithelial, endothelial, and mesenchymal cells. Thus, xenograft models comprising various tissues and cell types are of particular importance to evaluate the direct consequences of active infection on the relevant human cell types and to evaluate viral dissemination. Moreover, they are crucial for the evaluation of antiviral therapies to treat viral infections and prevent their further spread.

Modeling of active lytic infections in human tissue is still not well established for representatives of the alphaherpesvirus family, as only a few models have been described. In order to evaluate the replication of Varicella–Zoster Virus (VZV) in human tissues encompassing differentiated skin and T cells, SCID mice were engrafted with fragments of human skin as well as the fetal liver and thymus tissues, as these tissues play crucial roles in VZV infection. Afterward, xenografts were directly infected with wild-type or mutant VZV strains to elucidate the impact of ORF 47 and ORF66 gene products for VZV replication in vivo [152].

Skin xenograft models confirmed that infected T cells can release infectious virions in vivo [82,134,135]. Notably, VZV infection also induced slow progression of typical skin lesions and was associated with a robust innate immune response of epidermal cells in the skin [153]. Moreover, VZV-based dampening of host IFN-α production was observed in the infected skin cells, which subsequently blocked the recruitment of inflammatory cells to sites of virus replication [95]. These skin graft models helped to resolve the mechanisms of T cell-based virus transmission through the circulation to the skin and implicated in the viral blockade of the innate immune response.

Humanized models that incorporate relevant human tissues have been frequently applied to study HCMV infection. A pioneering study in this field was performed in SCID-huThy/Liv mice, revealing that HCMV can replicate in thymic epithelial cells [136]. The same experimental system was later used to test new antiviral drugs against HCMV (reviewed in [154]). A simpler model that is suited for larger scales was developed by Bravo and colleagues. By introducing HCMV-infected human fibroblasts onto a gelatin matrix and subsequently implanting these constructs subcutaneously into SCID mice [98], the authors of the study developed a model that facilitated the straightforward and convenient assessment of novel antiviral drugs targeting HCMV in vivo [144]. Also, the above-described skin xenograft models were employed for studying HCMV infection in which skin tissue was transplanted subcutaneously into SCID mice. Briefly, the human skin graft was infected with HCMV by direct intraxenograft injection, resulting in acute lytic infection in various cell types of the xenograft. Moreover, virus dissemination to other areas of the xenograft was also observed, which could be controlled by treatments with antivirals. This model has the potential to study the course of HCMV infection in different cell types and investigate viral spread through complex tissue organization; moreover, it represents a platform for testing new antivirals [155].

Herpesvirus infections are known to cause a range of pathological conditions in combination with other diseases, and there have been numerous attempts to develop mouse models to study these implications. Among the severe complications encountered by AIDS patients, the development of retinitis has been linked to HCMV infection. In 1994, Epstein and colleagues established a mouse model to investigate this connection by implanting human fetal retinal tissue into immunodeficient (SCID) mice. The successful infection and replication of HCMV within the human retinal tissue provided a valuable platform to study HCMV replication in neural tissue and its impact on the retina. Additionally, this model allowed for the evaluation of the therapeutic potential of various antiviral agents, highlighting the importance of developing reliable and effective treatments to combat the devastating effects of HCMV-associated retinitis [156].

Similarly, there is a connection between hepatitis and HCMV infection that is well-documented in immunocompromised patients, especially those who have undergone liver transplantation. Moreover, a few reports indicate that HCMV infection can also contribute to the development of hepatitis in immunocompetent individuals [157]. To shed light on the influence of HCMV infection on the pathogenesis of hepatitis, primary human hepatocytes obtained from human livers were engrafted via splenic injection of SCID/albumin linked-urokinase type plasminogen activator (SCID/Alb-uPA) transgenic mice—a well-established model for engrafting human hepatocytes. Subsequently, these humanized mice were intraperitoneally infected with a clinical strain of HCMV, and the plasma HCMV titers were closely monitored. Notably, viral DNA was detectable up to day 11 post-infection and was effectively reduced upon ganciclovir treatment [97]. This pioneering in vivo model not only enables the study of acute HCMV infection in human hepatocytes but also offers the opportunity to assess the efficacy of antiviral drugs.

HCMV infection poses a significant threat to the success of organ transplantations. Several studies suggested an increased risk of transplant rejection linked to newly acquired HCMV infection or reactivation of latent HCMV infection due to the immunosuppressive environment required during the transplantation procedure [158,159]. In an effort to unravel the complex interplay between HCMV and immune rejection of the graft, HCMV-infected human internal mammary artery tissue was transplanted into the infrarenal artery of immunodeficient mice. One week later, human PBMCs were transferred intraperitoneally, resulting in the appearance of vascular lesions and infiltration of immune cells. The viral infection was followed by xenograft rejection, emphasizing the crucial role played by HCMV in the immune-mediated rejection of transplanted organs [160].

The absence of KSHV-induced lesions in HSC humanized mice, highlighted above, promoted the development of advanced approaches for modeling KS. In the first approach, human skin was transplanted into SCID mice. Notably, infection of these transplants resulted in the formation of KS-like lesions, highlighting the effectiveness of tissue xenograft murine models [96]. In two other studies, KS-like tumors formed in immunocompromised mice transplanted with KSHV-infected immortalized endothelial cells [16,17]. In an attempt to identify novel antiviral therapeutics, this cell system was used to establish an in vitro/in vivo screening and validation pipeline. This allowed the identification of novel compounds that impaired KSHV-induced lesions in the mouse [161,162], paving the way for developing specific treatments for KSHV-associated diseases.

### 4.4. Herpesvirus Infection in Graft Models with Infection-Permissive Tissues in the Presence of a Reconstituted Human Immune System

The tissue structure and organization are considered to play a role in the dissemination of herpesviruses throughout the body and therefore represent crucial factors in the pathogenesis of infections. A recent study by Wahl and colleagues presented humanized mouse models that take into account the tissue structure and microenvironment constituted by various cell types [35]. In one of the models, so-called ‘human lung only mice’ (LoM), pieces of the human fetal lung were subcutaneously transplanted in NSG mice. The structural features of the grafts strongly resembled the structural organization of normal human lung tissue, including ciliated epithelium, alveolar structures, blood vessels, and cartilage. These human lung grafts supported the replication of HCMV and allowed the measurement of HCMV gene expression during the lytic cycle in vivo. To investigate the immune response toward HCMV infection, the authors further combined the LoM and BLT models into the so-called BLT-L model. To this end, they first transplanted pieces of human fetal liver and thymus under the kidney capsule of NSG mice, subsequently implanted autologous lung tissue, and finally injected autologous liver-derived hematopoietic stem cells. This complex strategy accurately recapitulated hematopoietic cell populations inside and outside the lung transplants. Upon injection of different HCMV strains into the lung transplants, an HCMV-specific adaptive and humoral immune response was observed in mice, characterized by functional control over the replication of the virus. In addition, the BLT-L model enabled the monitoring of the production of affinity-matured antibodies, a significant advancement towards the testing of new vaccines and antiviral drugs, as well as the development of highly potent neutralizing antibodies against pathogenic viruses.

## 5. Conclusions and Perspectives

Humanized mouse models have been recognized as valuable tools in investigating viral infections, particularly in understanding their dissemination, establishment of latency, and reactivation in vivo. Through these models, the manipulation of key host factors and signaling pathways by the virus for persistence can also be elucidated. Moreover, several humanized mouse models successfully recapitulated the main clinical aspects of herpesvirus infections. For instance, these models enabled the mimicking of various phenotypes caused by EBV and HCMV infections, such as lymphoproliferation [126,147], rheumatoid arthritis [148], hemophagocytic lymphohistiocytosis [163], and reactivation provoked by treatments like G-CSF [125,138]. They have provided insights into the disease mechanisms and helped improve diagnosis and develop treatment approaches in the context of EBV and HCMV infections. However, further developments are necessary to improve their robustness and achieve more effective engraftment of additional cell types, such as endothelial and epithelial cells. Such improvements can be expected to provide relevant insights for the development of antiviral treatments and vaccines for herpesviruses.

The worldwide prevalence and burden of herpesvirus infections, along with high species specificity, were the main driving forces for the development of numerous murine models that allow the study of these viruses. These models have different complexities: classical wild-type mice for infections with susceptible human herpesviruses or murine homologous viruses, genetically modified mice permissive to human viruses, xenotransplanted mice with human blood cells, and advanced humanized mice providing both a reconstituted human immune system and infection-relevant human cells or tissues (Figure 1). Recent advances in these models have allowed the reflection of multiple aspects of herpesvirus infections, including infection establishment and viral spread in vivo, mechanisms of the antiviral immune response, development of infection-related pathologies, and testing new antivirals and vaccines.

While the progress in mouse genetic engineering allowed the rational development of new murine immunodeficient strains, humanized models still have certain limitations to overcome. In the future, increasing engraftment rates of human cells, establishing proper innate immune cell development and function, and enabling proper B cell maturation are crucial points to address. More recent advances in this field were achieved upon extending the level of humanization of mice by targeting genes encoding human cytokines M-CSF, IL-3, GM-CSF, TPO, and SIRPα into the respective mouse loci (MISTRG mice based on Rag2−/−IL2rg−/− immunodeficient mice) [121]. The introduction of these human genes essential for the proper development of the human immune system resulted in a high efficiency of human hematopoietic engraftment and robust development of diverse subsets of human innate immune cells. This is particularly true with myeloid cells, which were the limitation in previous models, making MISTRG mice promising models for various diseases in humans. Future developments might further improve the still-limited adaptive immune responses like low humoral immune responses.

Murine models have already made significant contributions to the herpesvirology field; however, there are still major challenges associated with their use. Apart from the general challenges of animal experimentation, this includes the need for specialized (surgical) techniques in certain models, which require advanced personnel training and working conditions. The complex experimental conditions and the immune reactions of the transplanted cells are often associated with an elevated mortality rate, which requires larger group sizes and increases costs. In addition, the use of human donor tissues increases experimental variability, due at least partly to differences in the quality of the grafted cells or tissues, the donor’s genetic background, age, and environmental factors that are difficult to define. In addition, the limited access to human tissues frequently forces studies to rely on small sample sizes.

Moreover, many xenotransplant models rely on the transfer of embryonal cells since they usually provide better grafting efficiencies. Thus, ethical debates have arisen concerning the use of fetal tissues in such models. Progress can be expected based on the advent of induced pluripotent stem cells, which have the potential to differentiate into all cell types of the human body. With the development of robust, cost-effective protocols, large-scale generation of differentiated human cells seems feasible in the future.

The inherent challenges of the humanized mouse models have fostered the development of improved multicellular in vitro models that support the investigation of virus infections. Organoid cultures derived from induced pluripotent or adult stem cells closely mimic the structure and function of the original organs [164]. They could offer a valuable alternative to model infectious diseases and bridge the gap between mouse models and humans. Furthermore, organoid cultures have already been used for studying various viruses [165,166,167,168,169,170,171]. In the context of herpesviruses, brain organoids have proven to be a valuable tool in studying the mechanisms of HSV-1 infection during both its lytic and latent phases [165,166], as well as investigating the effects of HCMV infection on neuronal development [172,173]. With further progress in organoid cultivation, organoid models for studying herpesvirus infections in other relevant tissues can be foreseen.

Despite certain limitations, humanized mouse models remain important tools for infection research. Herpesviruses are one of the most powerful and complex natural manipulators of the human immune system and act on different levels. Thus, while recent trends in disease modeling can contribute to the reduction of animal experiments, studying herpesvirus infections only on in vitro models is currently insufficient to address all the crucial aspects of pathogenesis. Further improvements in mouse strains, supported by predictions from in vitro models such as organs-on-a-chip [174], might provide new insights and tools in the ongoing fight against these viral infections.

## Figures and Tables

**Table 1:** Characteristics of human herpesviruses.

	Virus Names	Transmission Media	Tropism in Human Cells	Infection Associated Pathophysiology in Humans (Examples)	Animal Homologues (Examples)
**Alphaherpesvirinae**	HHV-1/ Herpes simplex virus (HSV)-1	Body fluids (e.g., saliva, mucus, wound fluid)	Epithelial cells (mucosa, skin), neurons	Cold sores, stromal keratitis, genital herpes, HSE, meningitis, eczema herpeticum, pneumonitis	Bovine herpesvirus 1 (BHV-1) Suid Herpesvirus 1 (SuHV-1)
	HHV-2/ Herpes simplex virus (HSV)-2	Body fluids (e.g., saliva, mucus, wound fluid)			
	HHV-3/ Varicella zoster virus (VZV)	Direct contact, aerosols, vesicular fluids	T cells, skin epithelial cells, neurons	Varicella (chickenpox), Zoster (shingles), postherpetic neuralgia, meningoencephalitis, myelitis, vasculopathy, keratitis, retinopathy, visceral and gastrointestinal disorders	Cercopithecine alphaherpesvirus 9 (CeHV-9) Simian varicella virus (SVV)
**Betaherpesvirinae**	HHV-5/ Cytomegalovirus (HCMV)	Body fluids (e.g., saliva, semen, breast milk, mucus, transfusions)	PBMCs (monocytes, CD34^+^ progenitor cells, dendritic cells), epithelial cells, endothelial cells, fibroblasts, smooth muscle cells	Congenital infections: mental retardation, hearing loss, miscarriage. Transplant patients: graft rejection, hepatitis, pneumonitis, retinitis, cardiovascular diseases	Murine cytomegalovirus (MCMV)
	HHV-6A/ Roseolovirus	Saliva, (congenital transmission in case of chromosomal integration of viral DNA)	CD4^+^ T cells, PBMCs	Exanthema subitum (HHV-6B), febrile seizures, encephalitis, hepatitis, colitis, pneumonitis, GVHD, graft rejection, myelitis, neurological disorders, and oncogenesis	Murine roseolovirus (MRV) Porcine roseolovirus (PRV)
	HHV-6B/ Roseolovirus				
	HHV-7/ Roseolovirus	Body fluids (e.g., saliva, breast milk)	CD4^+^ T cells	Exanthema subitem, encephalitis, meningitis, acutemyelitis, Guillain–Barré syndrome	
**Gammaherpesvirinae**	HHV-4/ Epstein-Barr virus (EBV)	Body fluids (e.g., saliva, semen, blood)	Epithelial cells, B cells	Infectious mononucleosis, Burkitt’s lymphoma, nasopharyngeal carcinoma, gastric carcinoma, T cell lymphomas	Murine gammaherpesvirus 68 (MHV-68) Rhesus monkey rhadinovirus (RRV)
	HHV-8/ Kaposi’s sarcoma-associated herpesvirus (KSHV)	Body fluids (e.g., saliva, mucus, genital secretion, semen, breast milk, blood)	B cells, endothelial cells, epithelial cells, keratinocytes, fibroblasts, dendritic cells, monocytes/macrophages	Kaposi’s sarcoma (KS), primary effusion lymphoma (PEL), multicentric Castleman’s disease (MCD)	

**Table 2:** Experimental mouse models for human herpesviruses.

Virus	Establishment of Model	Achievements/Features	Limitations/Challenges
***Human viruses productively infecting mice***			
HSV-1 HSV-2	Infection of wildtype or genetically modified mice via different infection routes	Latency Innate immunity Role of viral genes in vivo T cell activation	Lack of spontaneous reactivation Lack of human immune system
***Orthologue viruses in mouse as native host***			
MCMV	Infection of wildtype or genetically modified mice via different infection routes	Cellular tropism Congenital infection	Role of private genes Human immune response Immune evasion Route of infection
MHV-68	Infection of wildtype or genetically modified mice via different infection routes	Lymphocytes tropism Chronic infection and latency Innate immunity	Endothelial cell tropism Route of infection Differences in latency program Lack of KS lesions
MRV	Infection of wildtype neonates	T cell tropism	Latent infection Infection in adult animals

**Table 3:** Overview of humanized mouse models.

Model	Establishment of Model	Genetic Background	Features	Limitations	References (Examples)
hu-PBL	IP injection of human PBMCs	SCID, NOD-SCID, NSG, BRG	T cell engraftment	No multilineage hematopoiesis No primary immune response Development of early GvHD	[29]
hu-HSC	Injection of human CD34^+^ cells from cord blood or fetal liver	SCID, NOD-SCID, NSG, BRG, NRG	Multilineage hematopoiesis Primary immune response	No HLA restriction Inadequate innate immune system	[30]
SCID- hu Thy/Liv	Implantation of human fetal thymus and liver fragments	SCID	Multilineage hematopoiesis	Immature T cells Low myeloid cells repopulation No B cells	[31]
hu-BLT	Implantation of human fetal thymus, liver fragments, and human CD34^+^ cells from fetal liver	NOD-SCID, NSG, NRG	Multilineage hematopoiesis Primary immune response HLA T cells restriction Functional memory T cells	Human fetal tissue Development of late GvHD Lower myeloid cells repopulation Non-functional NK cells	[32,33]
Skin graft models	Subcutaneous implantation of human fetal or adult skin tissue	SCID, SCID-beige athymic nude, NOD-SCID, NSG, BRG	Resident human immune cells Modeling multiple skin-related diseases Drug screening Fast and simple readouts	Reduced vascularization Need for additional reconstruction of human immune response Human fetal tissue	[34]
LoM	Subcutaneous implantation of human fetal lung tissue	NSG	Vascularized human lung implants Human cytokines and chemokines	Few hematopoietic cells detected Human fetal tissue	[35]
BLT-L	Implantation of human fetal thymus, liver fragments, and human CD34^+^ cells from fetal liver Subcutaneous implantation of human fetal lung tissue	NSG	Multilineage hematopoiesis Primary immune response HLA T cells restriction Functional memory T cells Vascularized human lung implants Human cytokines and chemokines	Human fetal tissue	[35]

Abbreviations: HLA: Human Leukocyte Antigens; PBMC: Peripheral Blood Mononuclear Cells; GvHD: Graft versus Host Disease; SCID: severe combined immunodeficient mice; NOD-SCID: non-obese diabetic SCID mice; NSG: NOD-SCID−/−IL2rg−/− mice; NOG: NOD−/−IL2rg−/− mice, and BRG: Balb/c Rag2−/−IL2rg−/− mice. For more details of the mouse strains see text.

**Table 4:** Most commonly used mouse strains for the establishment of humanized mouse models.

Strain	Characteristics	References
**Athymic Nude ** NU(NCr)-Foxn1^nu^	T cell deficiency	[101]
**SCID** Prkdc^scid^	T and B cell deficiency	[30]
**SCID/beige** Lyst^bg^ Prkdc^scid^	T and B cell deficiency Reduced NK cell activity	[108]
**NOD-SCID** NOD Prkdc^scid^	T and B cell deficiency Phagocytic tolerance	[106,112]
**NOG/NSG** NOD-SCID IL2rg−/−	T, B, and NK cell deficiency Phagocytic tolerance	[118,119]
**NRG** NOD-Rag2−/−IL2rg−/−	T, B, and NK cell deficiency Phagocytic tolerance	[120]
**BRG** BALB/c-Rag2−/−Il2rg−/−	T, B, and NK cell deficiency Phagocytic tolerance	[114]
**MISTRG** M-CSFh/hIL-3/GM-CSFh/hSIRPah/hTPOh/hRAG2−/−IL2Rg−/−	T, B, and NK cell deficiency Phagocytic tolerance Expression of human cytokines	[121]

## Data Availability

Not applicable.
